# Verapamil-loaded supramolecular hydrogel patch attenuates metabolic dysfunction-associated fatty liver disease via restoration of autophagic clearance of aggregated proteins and inhibition of NLRP3

**DOI:** 10.1186/s40824-023-00342-5

**Published:** 2023-01-20

**Authors:** Do Kyung Kim, Daewon Han, Jeongyun Bae, Haeil Kim, Solji Lee, Jong-Seok Kim, Young-Gil Jeong, Jongdae Shin, Hwan-Woo Park

**Affiliations:** 1 Department of Anatomy, Konyang University College of Medicine, Daejeon, 35365 Republic of Korea; 2 Department of Cell Biology, Konyang University College of Medicine, Daejeon, 35365 Republic of Korea; 3 Myunggok Medical Research Institute, Konyang University College of Medicine, Daejeon, 35365 Republic of Korea

**Keywords:** Carboxymethyl pullulan, Transdermal delivery, Hydrogel, Metabolic associated fatty liver disease, Autophagic clearance, Inflammasome

## Abstract

**Background:**

Obesity, a serious threat to public health, is linked to chronic metabolic complications including insulin resistance, type-2 diabetes, and metabolic dysfunction-associated fatty liver disease (MAFLD). Current obesity medications are challenged by poor effectiveness, poor patient compliance, and potential side effects. Verapamil is an inhibitor of L-type calcium channels, FDA-approved for the treatment of hypertension. We previously investigated the effect of verapamil on modulating autophagy to treat obesity-associated lipotoxicity. This study aims to develop a verapamil transdermal patch and to evaluate its anti-obesity effects.

**Methods:**

Verapamil is loaded in biomimetic vascular bundle-like carboxymethyl pullulan-based supramolecular hydrogel patches cross-linked with citric acid and glycerol linkages (CLCMP). The investigation was then carried out to determine the therapeutic effect of verapamil-loaded CLCMP (Vera@CLCMP) on diet-induced obese mice.

**Results:**

Vera@CLCMP hydrogel patches with hierarchically organized and anisotropic pore structures not only improved verapamil bioavailability without modifying its chemical structure but also enhanced verapamil release through the stratum corneum barrier. Vera@CLCMP patches exhibit low toxicity and high effectiveness at delivering verapamil into the systemic circulation through the dermis in a sustained manner. Specifically, transdermal administration of this patch into diet-induced obese mice drastically improved glucose tolerance and insulin sensitivity and alleviated metabolic derangements associated with MAFLD. Furthermore, we uncovered a distinct molecular mechanism underlying the anti-obesity effects associated with the hepatic NLR family pyrin domain-containing 3 (NLRP3) inflammasome and autophagic clearance by the vera@CLCMP hydrogel patches.

**Conclusion:**

The current study provides promising drug delivery platforms for long-term family treatment of chronic diseases, including obesity and metabolic dysfunctions.

**Supplementary Information:**

The online version contains supplementary material available at 10.1186/s40824-023-00342-5.

## Introduction

Obesity and its associated disorders, including insulin resistance, metabolic inflammation, and type 2 diabetes, are major global public health issues that are linked to inflammatory mechanisms [1, 2]. Metabolic dysfunction-associated fatty liver disease (MAFLD) refers to a group of liver conditions that range from simple steatosis to fibrosis, and are frequently accompanied by a chronic low-grade inflammatory state in the liver, adipose tissue, and skeletal muscle, with increased production of proinflammatory mediators [3–5]. Although several treatments for obesity, such as weight loss surgery, liposuction surgery, pharmacological therapy, and reduced energy intake, have been tested [6–8], improved therapeutic strategies for obesity and MAFLD are urgently required because of the lack of long-term safety and efficacy or potential serious side effects associated with existing treatments.

Autophagy is an intracellular process that allows cells to degrade and recycle unnecessary intracellular components, such as lipid droplets, misfolded protein aggregates, or damaged organelles, to maintain liver metabolic homeostasis [9, 10]. Defects in the autophagic process inhibit the clearance of excessive lipids from lipid droplets, inclusion bodies, and toxic protein aggregates, which can trigger the development of MAFLD [11–13]. The ubiquitin-binding autophagy receptor p62/SQSTM1 (hereafter referred to as p62) recognizes polyubiquitinated substrates and anchors them to the autophagosome membrane, thereby promoting their autophagic degradation [14]. Under certain pathological conditions, insufficient autophagic clearance can impair protein turnover, contributing to the accumulation of p62 and ubiquitinated proteins [15, 16].

The NLR family pyrin domain-containing 3 (NLRP3) inflammasome is a large multiprotein complex comprising NLRP3, an apoptosis-associated speck-like protein containing a caspase recruitment domain (ASC), and caspase-1 [17]. The NLRP3 inflammasome autocatalytically activates caspase-1, which leads to the processing and secretion of the proinflammatory cytokines interleukin-1β (IL-1β) and IL-18 [18]. Obesity-mediated inflammation through the NLRP3 inflammasome appears to play an important role in MAFLD development [19]. Thioredoxin-interacting protein (TXNIP) is elevated in diabetes and obesity and has been linked to NLRP3 inflammasome activation [20–22].

Verapamil, a phenylalkylamine channel blocker, is used to treat high blood pressure, angina, and heart arrhythmia. We previously demonstrated that impaired intracellular Ca^2+^ homeostasis during obesity induces the accumulation of p62 and ubiquitinated protein inclusions in hepatocytes, and that treatment with L-type Ca^2+^ channel blockers, such as verapamil and nifedipine, protects mice from high-fat diet (HFD)-induced insulin resistance and hepatic steatosis [23–25]. Moreover, studies have shown that verapamil inhibits the TXNIP/NLRP3 pathways [26, 27]. However, Ca^2+^ channel blockers can be devastating due to their toxicity and require short dosing intervals due to their short half-life. Although Ca^2+^ channel blockers are rapidly absorbed from the gastrointestinal tract following oral administration, their bioavailability is poor owing to low solubility and rapid first-pass metabolism [28], prompting the development of drug delivery systems that provide various routes for verapamil administration into the body and allow for its controlled release to achieve the desired therapeutic effect in obesity-related diseases.

**Fig. 1:**
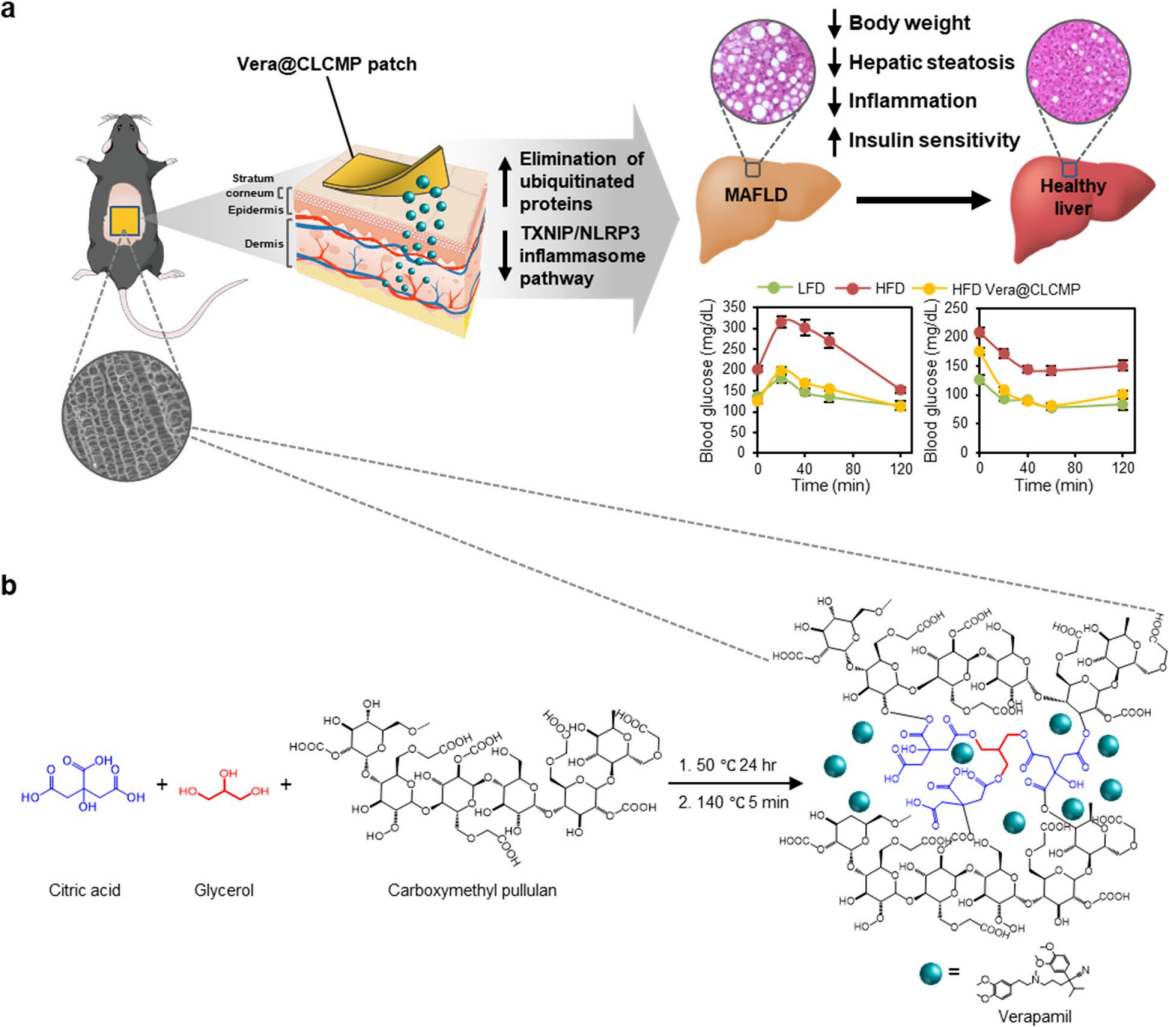
Synthesis and biomedical application of verapamil-loaded CLCMP (Vera@CLCMP) hydrogel patches. **A** Schematic illustration of Vera@CLCMP hydrogel patch attached to the mouse dorsal skin. Controlled release of the loaded verapamil in the superporous hydrogel system permeates to the stratum corneum or even deep dermis, thus reversing diet-induced obesity and insulin resistance. Vera@CLCMP patches attenuate obesity-induced metabolic dysregulation by improving autophagic clearance through regulation of CaMKII activity and NLRP3-inflammasome activation in hepatocytes. **B** Chemical structures schemes of Vera@CLCMP hydrogel patches

Since obesity is not an immediately life-threatening disease and requires long-term treatment, new therapeutic options must be safe, convenient, and tolerable. Transdermal drug delivery systems are an appealing therapeutic approach because of their numerous advantages, including painless application, excellent ease of administration, longer duration of action, persistence among patients, and reduced side effects, such as gastrointestinal damage [29]. In addition, transdermal drug delivery systems can avoid the first-pass effect of metabolism and allow sustained release of the drug. Hydrogels as promising candidates for drug delivery are porous hydrophilic crosslinking structures, which are formulated from natural or synthetic polymers. Their porous network structures enable the loading of therapeutic agents into the gel matrix of a hydrogel and their release at the desired speed. Owing to their excellent swelling capacity and biocompatibility, hydrogels are attractive materials for transdermal drug delivery patches. In this study, we report biomimetic vascular bundle-like supramolecular hydrogel patches composed of carboxymethyl pullulan cross-linked with citric acid and glycerol linkages (CLCMP), allowing sustained controlled release of verapamil while meeting adhesion requirements. A verapamil-loaded CLCMP (Vera@CLCMP) hydrogel patch was developed as a transdermal drug delivery system which enhances the drug’s bioavailability and sustains its release while reducing toxicity (Fig. 1a). We demonstrated prominent anti-obesity effects of Vera@CLCMP in mice. Our results suggest that sustained release of verapamil from Vera@CLCMP patches produces reversible autophagic clearance of aggregated proteins and inhibits the TXNIP/NLRP3 inflammasome pathway in in vitro and in vivo models of obesity using palmitate-treated human hepatoma HepG2 cell lines and diet-induced obese mice. Furthermore, transdermal administration of Vera@CLCMP significantly restored insulin sensitivity and reversed hepatic steatosis, indicating the promising implications for the treatment of obesity and its associated complications.

## Materials and method

### Materials

Pullulan was purchased from the Tokyo Chemical Industry (Tokyo, Japan). Isopropyl alcohol, citric acid, fluorescein isothiocyanate isomer I (FITC), sodium hydroxide (NaOH, 99.0%), monochloroacetic acid (ClCH_2_COOH), lithium aluminum hydride (LiAlH_4_), phosphate buffered saline (PBS, pH 7.4), and dialysis tubing cellulose membranes (Mw. cutoff = 14 K), (±)-verapamil hydrochloride, fatty acid-free bovine serum albumin (BSA), and palmitate were purchased from Sigma Aldrich (St. Louis, MO, USA). Glycerol, methanol, and ethanol were purchased from Duksan Chemicals (Seoul, South Korea). Immunoblotting and immunostaining were performed using antibodies against p62, NLRP3, cleaved caspase-3, phospho-CaMKII, TXNIP (Cell Signaling Technology, Danvers, MA, USA), p62 (Sigma-Aldrich), ubiquitin, CaMKII, caspase-1 (Santa Cruz Biotechnology, Santa Cruz, CA, USA), α-tubulin, β-actin (Developmental Studies Hybridoma Bank, Iowa City, IA, USA), and GAPDH (Aviva Systems Biology, San Diego, CA, USA). Triple-distilled and deionized water were used throughout the experiment.

### Synthesis and characterization of Vera@CLCMP patch

For CMP synthesis, 10 g of pullulan was completely dissolved in 40 mL of deionized water, and 12 mL of isopropyl alcohol was added. It suddenly coagulates and is dissolved again by sonication and stirring. After complete dissolution, 10 mL of 9 M NaOH was added to the mixture and heated at 70 °C for 15 min. The mixture was then treated with 10 mL of 3 M ClCH_2_COOH and 6 mL of isopropyl alcohol for 4 h at 70 °C. The color of the solution changed from light to dark brown. The same procedure for the addition of ClCH_2_COOH and NaOH was repeated and maintained at 70 °C for 4 h. Carboxymethylated pullulan was recovered by directly pouring the sample into 1 L of MeOH under magnetic stirring. The supernatant was decanted and washed several times with MeOH. Finally, the sample was dissolved in deionized water and dialyzed against 0.1 M HCl for 5 h. Subsequently, the impurities and unreacted reactants were purified by dialysis against deionized water for 5 days. Carboxymethylated pullulan was recovered with MeOH and dried in a vacuum concentrator. Different amounts of CMP, glycerol, and citric acid were then mixed at room temperature (RT). For example, 1 g of CMP, 0.1 g of glycerol, and 0.1 g of citric acid were dissolved in 20 mL of deionized water, and the air bubbles were removed by sonication. A 500 μL mixture solution was transferred into the silicon mold in each well. The CLCMP-loaded silicon mold was placed in a drying oven at 50 °C for 24 h and cured at 140 °C for 5 min. To load verapamil into the CLCMP patch, 200 μL of verapamil solution (25 mg/mL) was directly dropped onto the non-hydrated patch (0.2 g), kept in an oven at 50 °C for 5 h, and then vacuum dried.

FT-IR spectra were recorded using an ALPHA FT-IR spectrometer equipped with a platinum ATR (single reflection diamond ATR) from Bruker Optics. The spectra were recorded in the wavenumber range of 4000–400 cm^− 1^. In the FTIR study, pullulan, CLCMP, verapamil, Vera@pullulan, and Vera@CLCMP were measured by placing the samples on the surface of a diamond facet without any specific preparation of the specimens. 24 scans were performed for each sample.

For the swelling studies, the swelling ratio of the CLCMP hydrogel composites was determined using the following equations:

*Swelling ratio* = $$\frac{W_s-{W}_d}{W_{d.}}$$

where W_s_ and W_d_ represent the weight of the CLCMP hydrogel patches after swelling in deionized water and the weight of dehydrated CLCMP hydrogel patches after swelling, respectively.

The morphology of the CLCMP patch was analyzed using a SEM (SNE-4500 M, SEC Co., Korea) with a working voltage of 10 kV. The CLCMP patches were soaked in deionized water for 24 h, and the water was removed. The water-swollen CLCMP patches were frozen at − 80 °C for 1 h and dried without centrifugation using a vacuum concentrator. Vacuum-dried CLCMP patches were attached to the sample holder using carbon tape.

The thermal behavior of pullulan and CLCMP was measured using thermogravimetric analysis (TGA-50, Shimadzu) and differential scanning calorimetry (DSC-50, Shimadzu). The samples were recorded in the temperature range of 20–450 °C at a heating rate of 10 °C/min.

The chemical composition of the films was characterized by XPS using a commercial VG Microtech Multilab ESCA 2000 with a CLAM MCD detector and Al Kα radiation (1486.6 eV) operating at 1 × 10^− 8^ Torr. Survey scans were obtained in the range of 0–1400 eV, with an energy step of 1.0 eV, and a pass energy of 100 eV.

### Cell culture and treatments

Human hepatoma HepG2 cells were cultured in Dulbecco’s modified Eagle’s medium (DMEM; Welgene, Korea) supplemented with 10% fetal bovine serum (FBS; Welgene), 100 U/mL penicillin, and 100 μg/mL streptomycin. All cultures were maintained in a humidified atmosphere containing 5% CO_2_ at 37 °C. For palmitate treatment, the cells were incubated in the presence of palmitate/BSA as described previously [30]. Identical volumes of 10% fatty acid-free BSA solution were used as vehicle controls.

### Animal experiments

Eight-week-old male C57BL/6 mice were purchased from Samtako (Seoul, Korea). All mice were maintained under controlled temperature (21–24 °C) and humidity (50 ± 5%) with a 12 h light/dark cycle and were allowed free access to water and standard rodent diet (LFD) or HFD (60% fat, Research Diets, New Brunswick, NJ, USA) for 11 weeks. Mice were anesthetized and shaved on the dorsal side 1 d prior to the application of the transdermal patches. Pullulan and CLCMP-based patches, respectively, were applied to the dorsal skin (approximate area 1.5 × 1.5 cm) of mice thrice per week for 2 weeks and once per week for 2 weeks. Blood samples, liver tissues, and epididymal white adipose tissues were collected after the mice were sacrificed for molecular analysis.

### Cytotoxicity assay

HepG2 cell viability was evaluated using a WST-8 assay kit (Daeil Lab Service, Korea) according to the manufacturer’s instructions. HepG2 cells were briefly plated in 96-well plates at 1 × 10^4^ cells per well and treated with the indicated concentrations of verapamil dissolved in PBS, Vera@pullulan, and Vera@CLCMP patches for 24 h. Subsequently, 10 μL of the WST-8 reagent was added to each well and incubated for 30 min at 37 °C in a 5% CO_2_ incubator (Thermo Scientific, Waltham, MA, USA). Absorbance was measured using an Epoch2 microplate reader (Bio-Tek Instruments, Winooski, VT, USA) at a wavelength of 450 nm.

### Flow cytometry for apoptosis

HepG2 cells were plated in a 12-well plate at 4 × 10^5^ cells/well for 12 h. After incubation for 24 h with verapamil dissolved in PBS, Vera@pullulan, and Vera@CLCMP patches at the indicated concentrations, the adherent and floating cells were collected, washed with cold PBS, and stained using the Dead Cell Apoptosis kit with annexin V-FITC and propidium iodide (Thermo Fisher Scientific, Waltham, MA, USA). Data was analyzed using a CytoFLEX benchtop flow cytometer (Beckman Coulter, Fullerton, CA, USA).

### Glucose tolerance test and insulin tolerance test

Glucose tolerance tests (GTT) and insulin tolerance tests (ITT) were performed as previously described [25]. For the GTT, mice were injected intraperitoneally with D-glucose (1 g kg^− 1^ body weight) after 6 h of fasting. For ITT, mice were injected intraperitoneally with insulin (0.65 U kg^− 1^ body weight) after 6 h of fasting. At the indicated time points, blood was drawn from the tail and blood glucose was measured using an Accu-Chek blood glucose monitoring system (Roche Diagnostics, Indianapolis, IN, USA).

### Biochemical measurements

Blood samples were collected and allowed to clot for 30 min at RT, and then centrifuged at 3000 rpm for 30 min to collect the serum. Serum aminotransferase (ALT), aspartate aminotransferase (AST), and alkaline phosphatase (ALP) activity were measured using ALT, AST, and ALP activity assay kits (BioVision, Minneapolis, MN, USA), respectively.

### In vivo skin permeation and biodistribution studies

To visualize the location of verapamil after topical administration, FITC-conjugated verapamil was prepared using the following procedure: 0.1 mmol of verapamil dissolved in 1 mL of DMF, 0.2 mmol of LiAlH_4_ as catalysis dissolved in 1 mL of diethyl ether, and 20 μL H_2_O were stirred into 10 mL vials for 1 h at RT. The product was precipitated with excess diethyl ether and dried under flowing N_2_ gas. The aminated verapamil dissolved in 1 mL of EtOH was complexed with 0.1 mmol of FITC dissolved in 1 mL of EtOH. FITC-verapamil was fully dried using a speed vacuum concentrator (Operon, Korea) until further use.

To observe the skin cross-section, mice were anesthetized and shaved, and Vera@CLCMP-FITC patches were applied to the dorsal skin for 3 and 24 h. The collected skin tissues were embedded in an OCT compound (Leica Microsystems, Germany) and frozen at − 20 °C. Frozen skins were sectioned at a thickness of 10 μm using a cryostat microtome (Leica Microsystems). The sections were mounted onto glass slides with a mounting medium containing DAPI (Prolong Gold, Thermo Fisher Scientific). FITC fluorescence in the skin sections was observed using an epifluorescence-equipped microscope (DM2500, Leica, Germany).

For biodistribution studies, the dorsal skin of hairless mice was treated with a Vera-FITC@CLCMP patch or a CLCMP patch. Anesthesia was visualized using an in vivo imaging system (FOBI; Cellgentek, Korea) at specified times after treatment. After 1 day of imaging, major organs (heart, liver, spleen, kidney, lung, and pancreas) were collected from the mice, and the integrated fluorescence intensity of FITC was measured using NEOimage software (Cellgentek, Seoul, South Korea).

### Histology

Liver and white adipose tissues were prepared for histological analysis and stained with hematoxylin and eosin (H&E) and Oil Red O as previously described [25]. Briefly, the liver and white adipose tissues were fixed in neutral buffered formalin, embedded in paraffin, and stained with H&E. Paraffin-embedded sections were deparaffinized, rehydrated, and subjected to antigen retrieval. Endogenous peroxidase activity was quenched with 3% hydrogen peroxide. After non-specific antigens were blocked, the sections were incubated overnight at 4 °C with anti-p62 and anti-NLRP3 antibodies, followed by incubation with biotinylated anti-rabbit secondary antibodies (Vector Laboratories, Burlingame, CA, USA). Antibodies were visualized using streptavidin-HRP (BD Biosciences, San Diego, CA, USA) and 3, 3′-diaminobenzidine (Sigma-Aldrich). The sections were counterstained using hematoxylin. The OCT-embedded frozen liver tissues were sectioned and stained with Oil Red O (Sigma-Aldrich). The samples were observed under a light microscope (Leica, Wetzlar, Germany).

### Solubility fractionation

Solubility fractionation was performed as previously described [24]. Liver tissues and HepG2 cells were lysed in lysis buffer (20 mM Tris-Cl pH 7.5, 150 mM NaCl, 1 mM EDTA, 1 mM EGTA, 2.5 mM NaPPi, 1 mM β-glycerophosphate, 1 mM Na_3_VO_4_, and protease inhibitor cocktail) containing 1% Triton X-100. Lysates were centrifuged at 18,000×g for 15 min at 4 °C. The resulting pellet and supernatant fractions were defined as the Triton X-100-insoluble and Triton X-100-fractions, respectively. The pellets were resuspended in a lysis buffer containing 2% sodium dodecyl sulfate (SDS). The supernatants and resuspended pellets were boiled in SDS sample buffer and subjected to SDS-polyacrylamide gel electrophoresis (PAGE) and immunoblotting.

### Immunoblotting

Liver tissue and HepG2 cells were lysed for 20 min on ice in a radioimmunoprecipitation assay buffer containing a complete protease inhibitor cocktail (Roche), followed by centrifugation at 18,000×g for 15 min at 4 °C. The total protein concentration was measured using a bicinchoninic acid (BCA) protein assay (Thermo Fisher Scientific, Waltham, MA, USA). Lysates were boiled in 1× SDS Laemmli sample buffer for 5 min, separated by SDS-PAGE, and transferred to polyvinylidene fluoride membranes (Merck Millipore, Darmstadt, Germany), which were subsequently blocked with 5% nonfat milk powder (Bio-Rad, Hercules, CA, USA). The blots were probed with primary antibodies against ubiquitin, p62, phospho-CaMKII, CaMKII, NLRP3, caspase-1, TXNIP, α-tubulin, β-actin, and GAPDH. The blots were then washed and incubated with the appropriate horseradish peroxidase-conjugated secondary antibodies. After washing, the protein bands were detected by enhanced chemiluminescence using the SuperSignal West Femto chemiluminescent substrate (Thermo Scientific, Rockford, IL, USA). ImageJ software (National Institutes of Health, USA) was used to calculate the protein band intensities.

### Immunocytochemistry

The HepG2 cells were cultured on glass coverslips in 24-well plates. After treatment, the cells were fixed with 4% paraformaldehyde (pH 7.4) for 15 min at RT and permeabilized with methanol at − 20 °C. The cells were then blocked in a blocking solution for 1 h at RT and incubated with anti-p62, anti-ubiquitin, and anti-cleaved caspase-3 antibodies at 4 °C in a humidified chamber. The next day, the cells were stained with Alexa Fluor 488-conjugated secondary antibody or Alexa Fluor 549-conjugated secondary antibody (Thermo Scientific) for 1.5 hrs and counterstained with DAPI (Thermo Scientific). Fluorescent images were obtained using a laser scanning confocal microscope (LSM 700; Carl Zeiss, Jena, Germany).

### Quantitative real-time PCR

The total RNA was isolated from liver tissue homogenates using TRIzol reagent (Takara, Shiga, Japan), according to the manufacturer’s instructions. Complementary DNA was synthesized from RNA using a cDNA synthesis kit (BioFact, Daejeon, Korea). Relative gene expression was determined using SYBR Green qPCR Master Mix (BioFact) on a QuantStudio 3 real-time PCR system (Life Technologies, Carlsbad, CA, USA). The fold change in gene expression compared to LFD-fed mice was calculated using the comparative threshold cycle (C_t_) method, which was normalized to mouse *Gapdh*. The primers used in this study are listed in Additional file 1 Supplementary Table 1.

### Statistical analysis

Results are presented as mean ± standard error of the mean (SEM). The data presented in the figures is representative of at least three independent experiments unless otherwise stated. The significance of the differences between the two experimental groups was determined using a two-tailed Student’s *t*-test. Multiple comparisons were conducted using one-way analysis of variance (ANOVA) followed by Tukey’s *post-hoc* test. Differences were considered statistically significant at *p* < 0.05 (*p < 0.05, **p < 0.01, ***p < 0.001).

## Results

### Synthesis and characterization of Vera@pullulan and Vera@CLCMP patches

**Fig. 2:**
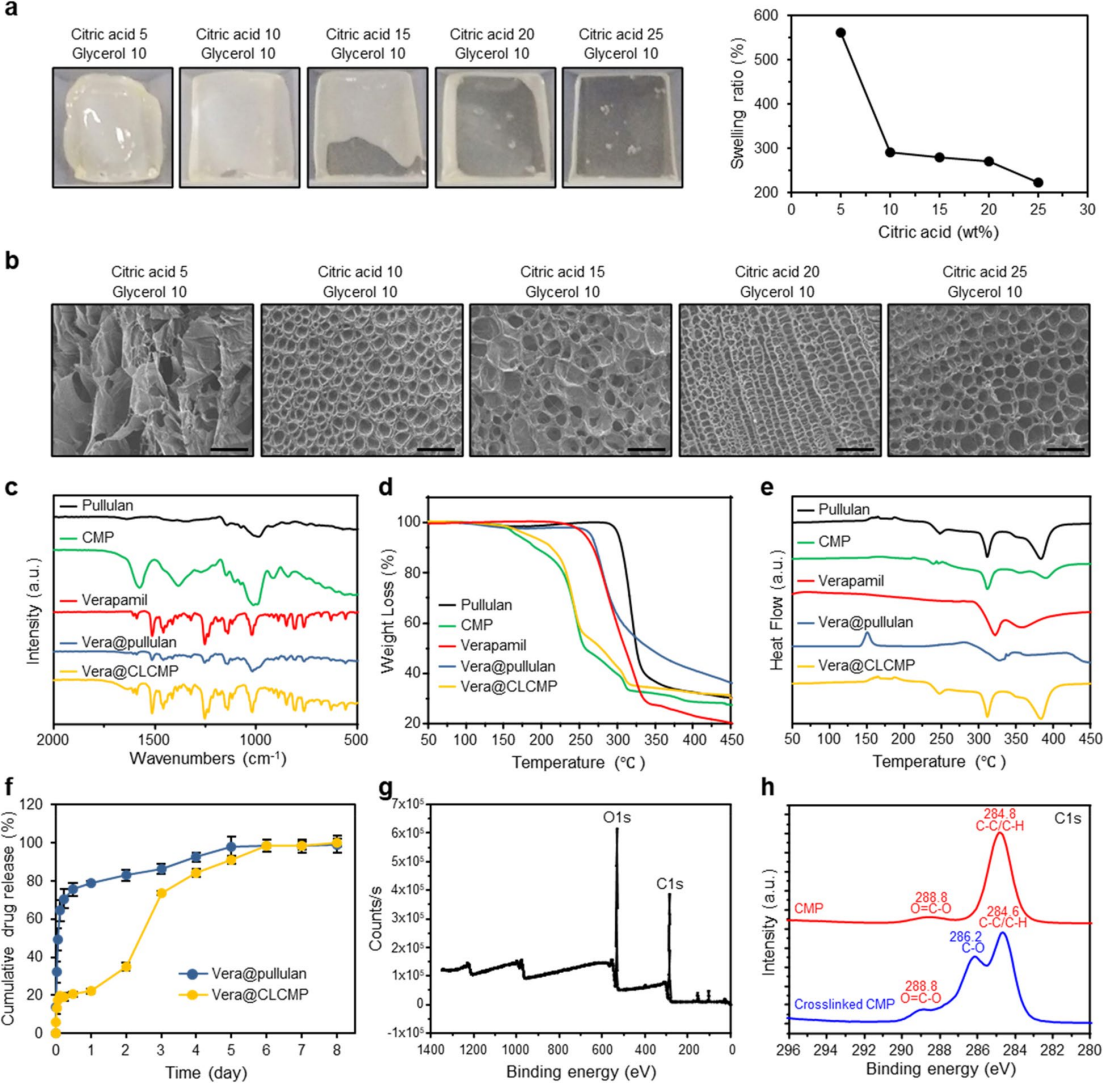
Characterization of pullulan patches containing verapamil (Vera@pullulan) and Vera@CLCMP patches. **A** Photographs of the Vera@CLCMP and swelling ratio depending on adding amount of citric acid. **B** SEM micrographs of the freeze-dried samples of 5–25 wt% citric acid and 10 wt% glycerol. Scale bar, 100 μm. (**C**) FT-IR spectra of pullulan, CLCMP, verapamil, Vera@pullulan, and Vera@CLCMP. **D** Thermogravimetric analysis (TGA) curves and (**E**) Differential scanning calorimeter (DSC) thermograms of pullulan, CLCMP, verapamil, Vera@pullulan, and Vera@CLCMP. **F** In vitro accumulated free verapamil release from Vera@pullulan and Vera@CLCMP patch in PBS at 37 °C. **G, H** Full range of X-ray photoelectron spectroscopy (XPS) spectra of CLCMP and XPS spectra of C-C/C-H, O=C-O and C-O of CMP and CLCMP. Data is shown as mean ± SEM

The esterification reaction was performed using a crosslinking reaction between citric acid, glycerol, and carboxymethyl pullulan (CMP) [31, 32]. The synthesis and Vera@CLCMP patch are illustrated in Fig. 1b. The esterification of the hydroxy group (−OH) of glycerol and CMP with the cyclic anhydride of citric acid produces a novel carboxylate group (COO-) which forms an intramolecular anhydride moiety with a neighboring carboxylate group [33]. Since citric acid esterification occurs in the solid state at high temperatures, CMP crosslinking was performed at 140 °C for 5 min. Figure 2a shows photographic images of CLCMP patches prepared in a silicon mold at 50 °C for 24 h. The CMP crosslinked with 5 wt% citric acid and 10 wt% glycerol was rigid, fragile, and deformed, losing its flat shape due to weak crosslinking. The flexibility of CLCMP patches, evaluated by varying the citric acid concentrations (5–25 wt%) from their dry to swollen state, increased linearly with increased citric acid concentration. The superabsorbent CLCMP hydrogel has elasticity that induces pore expansion in aqueous media, resulting in water absorption that can cause it to increase by 220–570 times of its dehydrated size (Fig. 2a). As expected, the increase in size of the CLCMP hydrogels was inversely proportional to their citric acid and glycerol concentrations (Additional file 1 Fig. S1a) [34]. Figure 2b and Additional file 1 Fig. S1b present scanning electron microscope (SEM) images of the freeze-dried CLCMP patches. Surprisingly, when compared to other CLCMP compositions, the CLCMP sample comprising 20 wt% citric acid and 10 wt% glycerol exhibited a supramolecular three-dimensional network structure with regularity and uniformity.

Figure 2c depicts the FTIR spectra of pullulan, CLCMP, verapamil, and Vera@CLCMP (Additional file 1 Fig. S2). The smaller peaks near 2655 and 2544 cm^− 1^ are characteristic of the H-bonded dimer of O-H stretching originating from the carboxylic group of CMP. The carbonyl (C=O) stretching frequency of the dimer was observed at approximately 1728 cm^− 1^. The peak at 3337 cm^− 1^ is attributed to anhydroglucose unit O-H stretching, while that at 2925 cm^− 1^ is attributed to C-H stretching. The symmetric carboxylate group (COO-) was responsible for the peak at 1416 cm^− 1^. Moreover, the three peaks near 756, 876, and 939 cm^− 1^ were characteristic of D-glycosidic bonds [35]. These results imply that characteristic peaks of CLCMP is consistent with previous reports [36].

Thermogravimetric analysis (TGA) (Fig. 2d) and differential scanning calorimetry (DSC) analysis (Fig. 2e) were performed to investigate the thermal properties of the pullulan and CLCMP patches. Approximately 3% of pullulan weight loss from 24 to 200 °C could be attributed to the dehydration of adsorbed moisture. Furthermore, a secondary weight loss appeared in the temperature range 300–500 °C, with a decomposition temperature (T_d_) of 282 °C, indicating pullulan chain degradation [37]. Rapid weight loss began, with 75% loss at 215–335 °C. The first weight loss associated with water dehydration was 1.5% below 150 °C, and the secondary weight loss associated with CLCMP thermal degradation was 68%. A similar thermal behavior was observed in Vera@CLCMP, because a very small amount of verapamil was incorporated into the CLCMP patch and did not significantly affect its thermal behavior. However, Vera@CLCMP presented approximately 3% less weight loss than CLCMP alone. Later, weight loss in the range of 200–416 °C corresponded to the evaporation of unreacted glycerol and the thermal degradation of CLCMP. After loading verapamil into the CLCMP patch, the T_d_ of Vera@CLCMP increased from 381 °C to 390 °C (Fig. 2d). These results indicate that, compared to the control patch, a T_d_ peak shift after loading pullulan and CLCMP patches with verapamil, indicating no chemical interaction between verapamil and the formulation excipients.

The release profile of the Vera@CLCMP patch in 1 day is consistent with the diffusion model (Fig. 2f) [25]. Similar to the diffusion model, a typical rate-determining step appeared after 1 d and lasted until 6 d. When the rate of absorption is greater than the rate of dissolution, a solid drug dissolved in a solvent may fit the dissolution model. The dissolution model is more suitable when the drug has is hydrophobic and poorly soluble. As verapamil is soluble in the aqueous phase, the release profile of Vera@pullulan should be similar to the diffusion model, which it is. However, the release profile of the Vera@CLCMP patch showed a model shift from the diffusion model to the dissolution model after day 1. The model shift during the release period is clear evidence that the CLCMP patch comprised CMP, glycerol, with citric acid existing as a highly crosslinked hydrogel. After 6 days, the cumulative verapamil release approached approximately 95%. The dissolution rate of Vera@CLCMP in this study is an important parameter in designing a proper transdermal delivery system, thus indicating an appropriate release rate over 6 days.

The CMP and CLCMP patches were further analyzed by XPS. Figure 2g shows a wide-range XPS spectrum of the CLCMP patch. The presence of covalent bonds between CMP, citric acid, and glycerol was confirmed by comparing the CMP and CLCMP high-resolution C1s XPS spectra. The presence of carbon is clearly demonstrated by the symmetric peak at 286.1 eV, and that of oxygen indicated by the peak at 531.1 eV. As shown in Fig. 2h, the C1s peak of CMP was deconvoluted into two peaks with binding energies of 284.8 °(C-C/C-H) and 288.8 (O=C-O). Meanwhile, the C1s peak of the CLCMP patch was deconvoluted into three peaks, appearing at 284.6 (C-C/C-H), 286.2 (C-O), and 288.8 (O=C-O). The C-O peak from glycerol appeared at 286.2°, consistent with the FTIR analysis.

### In vitro and in vivo toxicity of Vera@pullulan and Vera@CLCMP patches

**Fig. 3:**
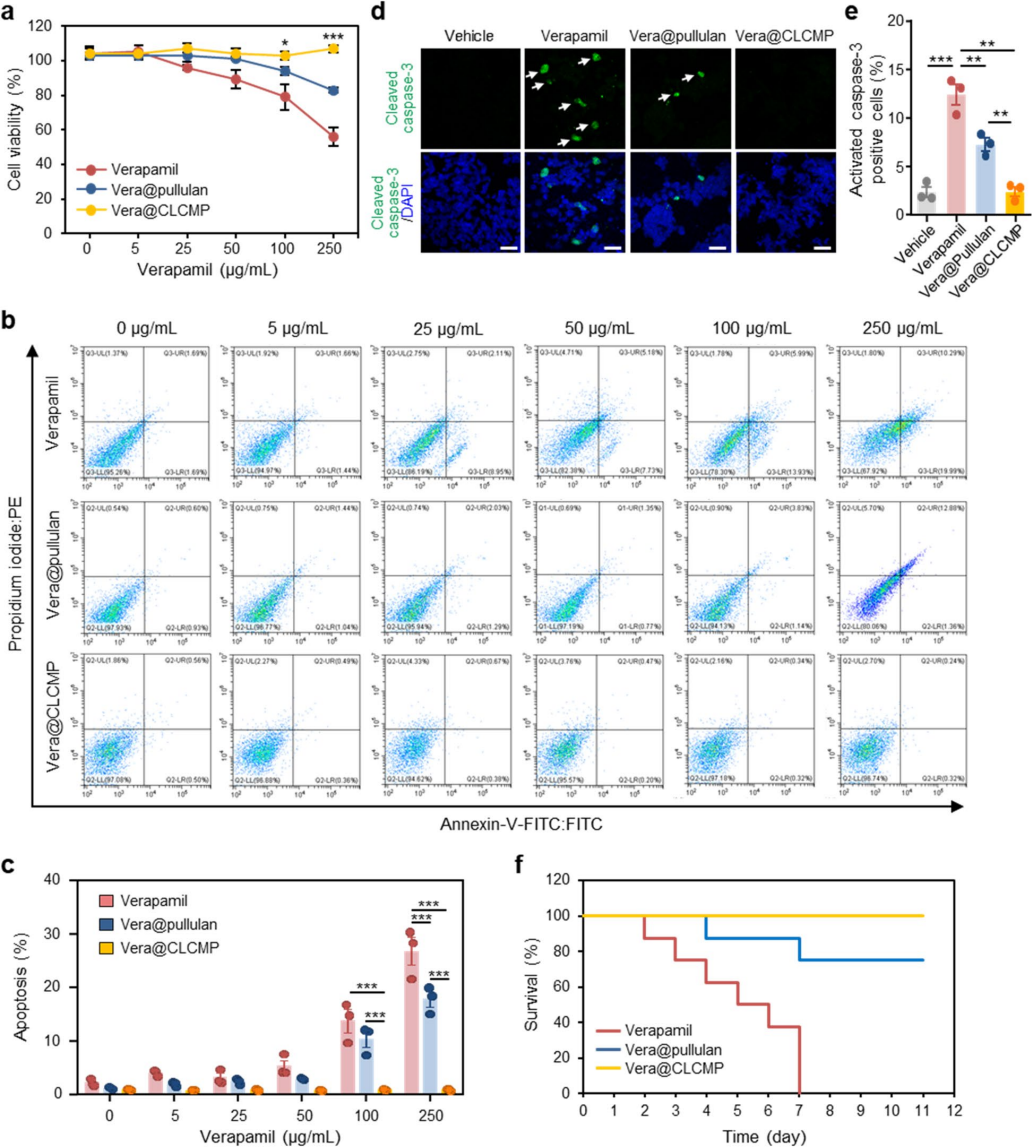
In vitro and in vivo toxicity of Vera@pullulan and Vera@CLCMP patches. a-c HepG2 cells were treated with the indicated concentration of verapamil dissolved in PBS, Vera@pullulan, and Vera@CLCMP patches, respectively, for 24 hrs. **A** Cell viability was measured by the WST-8 assay. **B** HepG2 cells were stained with annexin V-FITC and PI and then analyzed for apoptosis by flow cytometry. The percentage of apoptotic HepG2 cells are shown in (**C**). **D** Immunofluorescence staining for cleaved caspase-3 (green) in HepG2 cells treated with 250 μg/mL verapamil, 250 μg/mL Vera@pullulan, 250 μg/mL Vera@CLCMP, or PBS (vehicle) for 24 hrs. Nuclei were stained with DAPI (blue). White arrows show cleaved caspase-3 positive cells. Scale bar, 40 μm. **E** Caspase-3 activity was quantified. **F** Kaplan–Meier survival curve of mice treated daily with verapamil (50 mg/kg intraperitoneal [i.p.]), 50 mg/kg Vera@pullulan, and 50 mg/kg Vera@CLCMP patches, respectively, on day 0 (*n* = 6–14). Data is shown as mean ± SEM. *p < 0.05; ***p < 0.001 (One-way ANOVA, followed by Tukey’s test)

To compare the in vitro cytotoxicity of verapamil, Vera@pullulan patches, and Vera@CLCMP patches, we used the WST-8 assay to assess the viability of HepG2 cells treated with different concentrations of free verapamil, Vera@pullulan patches, and Vera@CLCMP patches for 24 h. Vera@CLCMP patches exhibited very low cytotoxicity up to an equivalent concentration of verapamil (250 μg/mL). However, treatment with free verapamil or Vera@pullulan patches resulted in cytotoxicity at high concentrations (Fig. 3a). We then used flow cytometry to look at the apoptotic effects of free verapamil, Vera@pullulan, and Vera@CLCMP in HepG2 cells 24 h after treatment, using annexin V-FITC and propidium iodide staining. Treatment with free verapamil or Vera@pullulan for 24 h significantly induced apoptosis in a dose-dependent manner, whereas Vera@CLCMP treatment did not affect apoptosis (Fig. 3b, c). To further confirm these results, we evaluated the expression of the active form of caspase-3 using immunofluorescence staining. The number of active caspase-3-positive cells was significantly elevated in HepG2 cells treated with free verapamil or Vera@pullulan. However, there was no change in the number of active caspase-3-positive cells following treatment with Vera@CLCMP (Fig. 3d, e). Similarly, the WST-8 assay revealed no statistically significant differences in cell viability between the control cells and those treated with any concentration of CLCMP aqueous solution (Additional file 1 Fig. S3a). Annexin V-FITC and propidium iodide staining showed almost no apoptotic pattern in HepG2 cells treated with any concentration of the aqueous solution of CLCMP (Additional file 1 Fig. S3b, c).

Next, we evaluated the safety and toxicity of the application of Vera@pullulan and Vera@CLCMP patches to mouse dorsal skin. Control mice were intraperitoneally injected with 50 mg/kg verapamil. On day 7, 80% of the mice treated with Vera@pullulan patches were still alive, whereas all mice in the control group died (Fig. 3f). Interestingly, Kaplan–Meier survival analysis revealed a significant improvement in the survival of mice treated with Vera@CLCMP patches compared to those treated with Vera@pullulan patches (Fig. 3f). Collectively, these results suggest that Vera@CLCMP patches do not cause appreciable toxicity in vitro or in vivo*.*

### Vera@CLCMP patches improve diet-induced glucose tolerance and insulin sensitivity

**Fig. 4:**
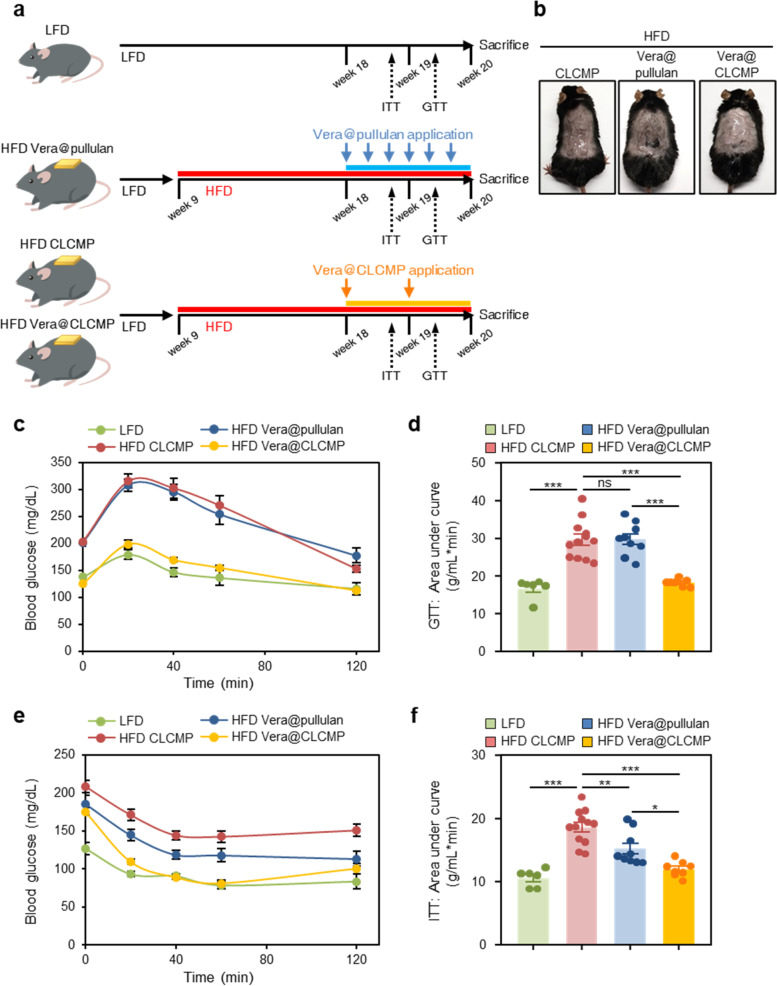
Vera@CLCMP patches improve glucose tolerance and insulin sensitivity. **A** Experimental design for in vivo treatment of high-fat diet (HFD)-induced obese mice. **B** Vera@pullulan or Vera@CLCMP patches were applied to the dorsal skin of mice in vivo. **C-F** Vera@pullulan or Vera@CLCMP patches were applied to the dorsal skin of C57BL/6 male mice kept on HFD. Low-fat diet (LFD)-fed mice of the same age were used as a negative control. Glucose tolerance test (GTT, **C**) and insulin tolerance test (ITT, **E**) were conducted using LFD-fed or HFD-fed mice treated with Vera@pullulan or Vera@CLCMP patch. Area under the curve was quantified from GTT (**D**) and ITT data (**F**). Data is shown as mean ± SEM. *p < 0.05; **p < 0.01; ***p < 0.001 (One-way ANOVA, followed by Tukey’s test)

To evaluate whether transdermal administration of Vera@pullulan and Vera@CLCMP patches affected glucose tolerance and insulin sensitivity in HFD-induced obese mice, male mice were randomly assigned to four groups: the normal group (LFD), in which mice were fed an LFD; the HFD group, in which mice were fed an HFD for 9 weeks; the HFD + Vera@pullulan patch group, in which mice were fed an HFD and were then treated with Vera@pullulan patches; and the HFD + Vera@CLCMP patch group, in which mice were fed an HFD and were then treated with Vera@CLCMP patches (Fig. 4a, b). Glucose and insulin tolerance test results indicated that HFD-fed mice exhibited impaired glucose tolerance and insulin resistance compared to LFD-fed mice (Fig. 4c-f). Interestingly, glucose tolerance was markedly improved in the HFD + Vera@CLCMP patch group but not in the HFD + Vera@pullulan patch group (Fig. 4c, e). The HFD + Vera@pullulan and HFD + Vera@CLCMP patch groups both had lower insulin resistance than the HFD group. Furthermore, the HFD + Vera@CLCMP patch group showed an even smaller area under the curve for glucose than the HFD + Vera@pullulan patch group (Fig. 4d, f). Together, these results clearly demonstrate that treatment with the Vera@CLCMP patch reduces HFD-induced glucose intolerance and insulin resistance.

### In vivo fluorescence imaging and distribution of Vera@CLCMP

**Fig. 5:**
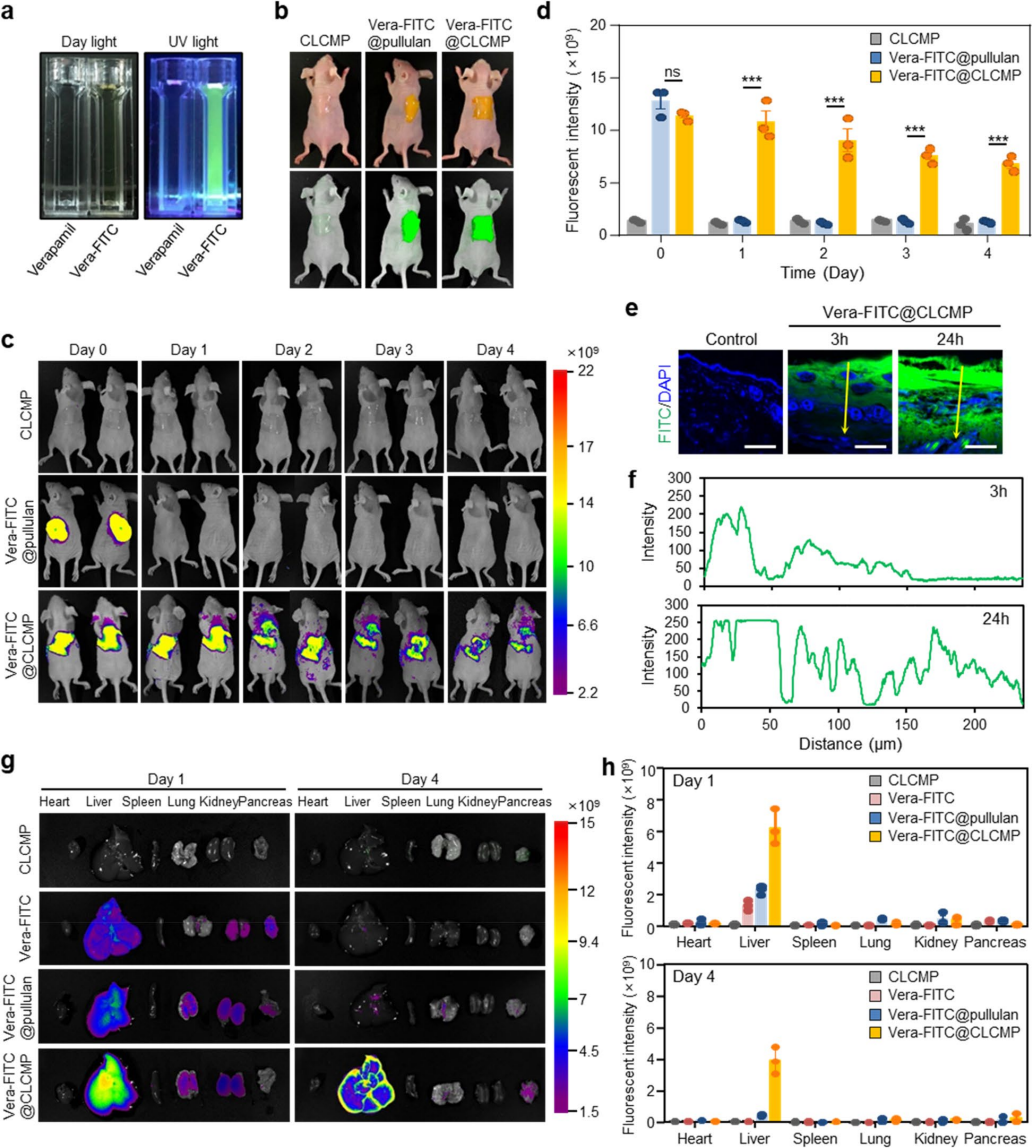
In vivo imaging and biodistribution of Vera-FITC@CLCMP patch in mice. **A** Photographs of verapamil and verapamil-FITC conjugate solutions under daylight and UV light for 365 nm excitation wavelength. **B** Vera-FITC@pullulan or Vera-FITC@CLCMP patches were applied to the dorsal skin of hairless mice. **C** Fluorescence imaging of mice at 1–4 days after treatment with CLCMP, Vera-FITC@pullulan, or Vera-FITC@CLCMP patches. **D** Fluorescence intensity was quantified. **E** Fluorescence imaging of histological sections of the dorsal skin of mice treated with Vera-FITC@CLCMP patch at indicated time points. Nuclei were stained with DAPI (blue). Scale bar, 100 μm. A line scan across the marked line is shown in (**F**). **G** Ex vivo fluorescence imaging of heart, liver, spleen, lung, kidney, and pancreas at 1 day and 4 days after treatment with CLCMP, Vera-FITC, Vera@pullulan, or Vera-FITC@CLCMP patches. **H** Fluorescence intensity of each organ was quantified. Data is shown as mean ± SEM. ***p < 0.001 (One-way ANOVA, followed by Tukey’s test)

We examined whether verapamil penetrated the skin barrier by using Vera@CLCMP patches. To trace verapamil, we conjugated fluorescein isothiocyanate (FITC) to verapamil and incorporated it into the CLCMP patches (Additional file 1 Fig. S4a). Strong green fluorescence, derived from verapamil-FITC, was observed in water (Fig. 5a). Vera-FITC@CLCMP patches and Vera-FITC@pullulan patches were topically applied to the dorsal skin of hairless mice (Fig. 5b). In vivo fluorescence imaging showed that a FITC fluorescent signal of Vera-FITC@CLCMP patches lasted at least 4 days after application. However, the signal of Vera@pullulan patches did not last more than 1 day (Fig. 5c, d, and Additional file 1 Fig. S5). No FITC fluorescent signal above the autofluorescence level was detected in the dorsal skin of the control mice. Verapamil-FITC penetrated the stratum corneum and reached the dermis within 24 h of application, according to histological analysis of fixed dorsal skin from the application areas of Vera-FITC@CLCMP-treated mice (Fig. 5e, f). Ex vivo fluorescence imaging was conducted to investigate the biodistribution profiles after transdermal administration of Vera-FITC@CLCMP, transdermal administration of Vera-FITC@pullulan, or intraperitoneal administration of verapamil-FITC. After transdermal administration of Vera-FITC@CLCMP patches, verapamil-FITC (as indicated by fluorescence) remained in the liver for at least 4 days. However, after transdermal administration of Vera-FITC@pullulan patches or intraperitoneal administration of verapamil-FITC, FITC signals did not last more than 1 day in mouse livers (Fig. 5g, h).

### Vera@CLCMP inhibits palmitate-induced accumulation of protein inclusions

**Fig. 6:**
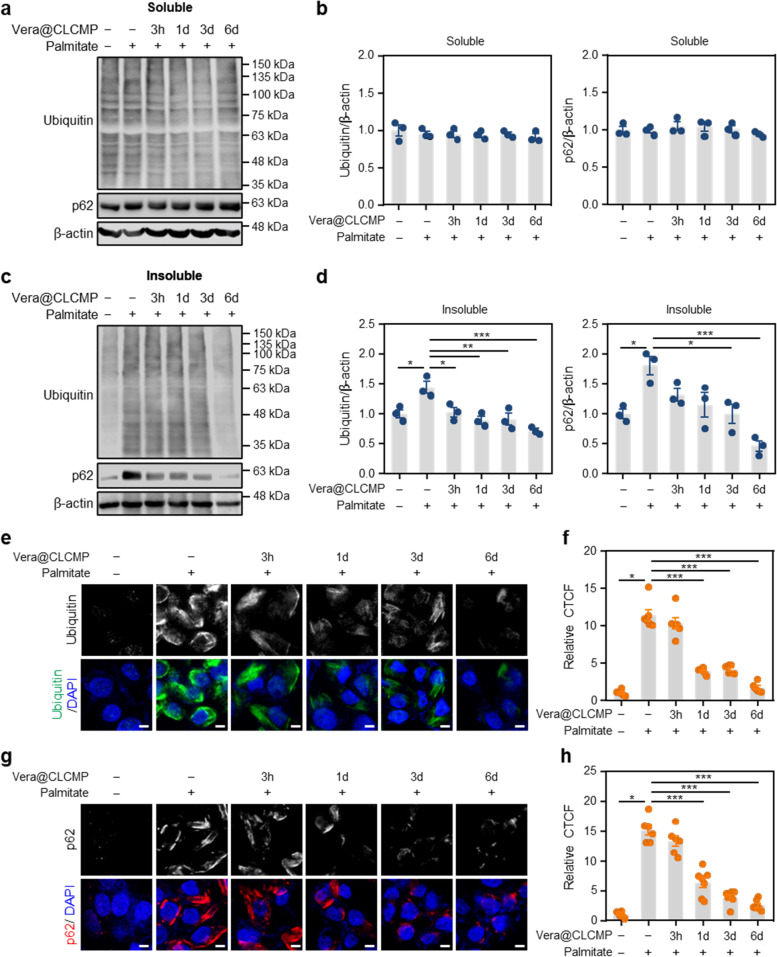
Vera@CLCMP improves palmitate-induced protein aggregation. **A-H** HepG2 cells were treated with 500 μM palmitate or BSA (vehicle) for 12 h in the presence or absence of the release medium and incubated with 38.35 μg/mL Vera@CLCMP at 37 °C for indicated periods. **A, C** Triton X-100-soluble and -insoluble fractions of cell lysates were immunoblotted with anti-ubiquitin and anti-p62 antibodies. β-actin served as a loading control. **B, D** Band intensities were quantified and normalized to control levels. **E, G** Immunofluorescence staining for ubiquitin (green) and p62 (red) in HepG2 cells with indicated treatments. Nuclei were stained with DAPI (blue). Scale bar, 5 μm. **F, H** Sizes of ubiquitin and p62 aggregates were quantified. Data is shown as mean ± SEM. *p < 0.05; **p < 0.01; ***p < 0.001 (One-way ANOVA, followed by Tukey’s test)

We previously reported the effect of Ca^2+^ channel blockers on autophagic degradation of protein aggregates in liver cells treated with saturated fatty acids [23–25]. Here, we examined whether Vera@CLCMP patches affected the saturated fatty acid-induced accumulation of protein inclusions. Immunoblot analysis indicated that no appreciable change was observed in the levels of ubiquitinated proteins and p62 in detergent-soluble fractions of HepG2 cells treated with the release medium of Vera@CLCMP patches at any time point (Fig. 6a, b). However, in a time-dependent manner, pretreatment with the release medium of Vera@CLCMP patches significantly palmitate-induced polyubiquitinated proteins and p62 in detergent-insoluble fractions of HepG2 cells (Fig. 6c, d). Using immunocytochemistry, we then investigated the effect of Vera@CLCMP patches on the formation of protein aggregates. The results showed that the release medium of Vera@CLCMP patches reduced the size of the polyubiquitinated aggregates in a time-dependent manner (Fig. 6e, f). Pretreatment with the release medium of Vera@CLCMP patches decreased the aggregation of palmitate-induced p62-positive protein (Fig. 6g, h). Thus, these findings suggest that sustained release of verapamil from Vera@CLCMP patches improves autophagic clearance.

### Vera@CLCMP patches reverse diet-induced obesity and hepatic steatosis

**Fig. 7:**
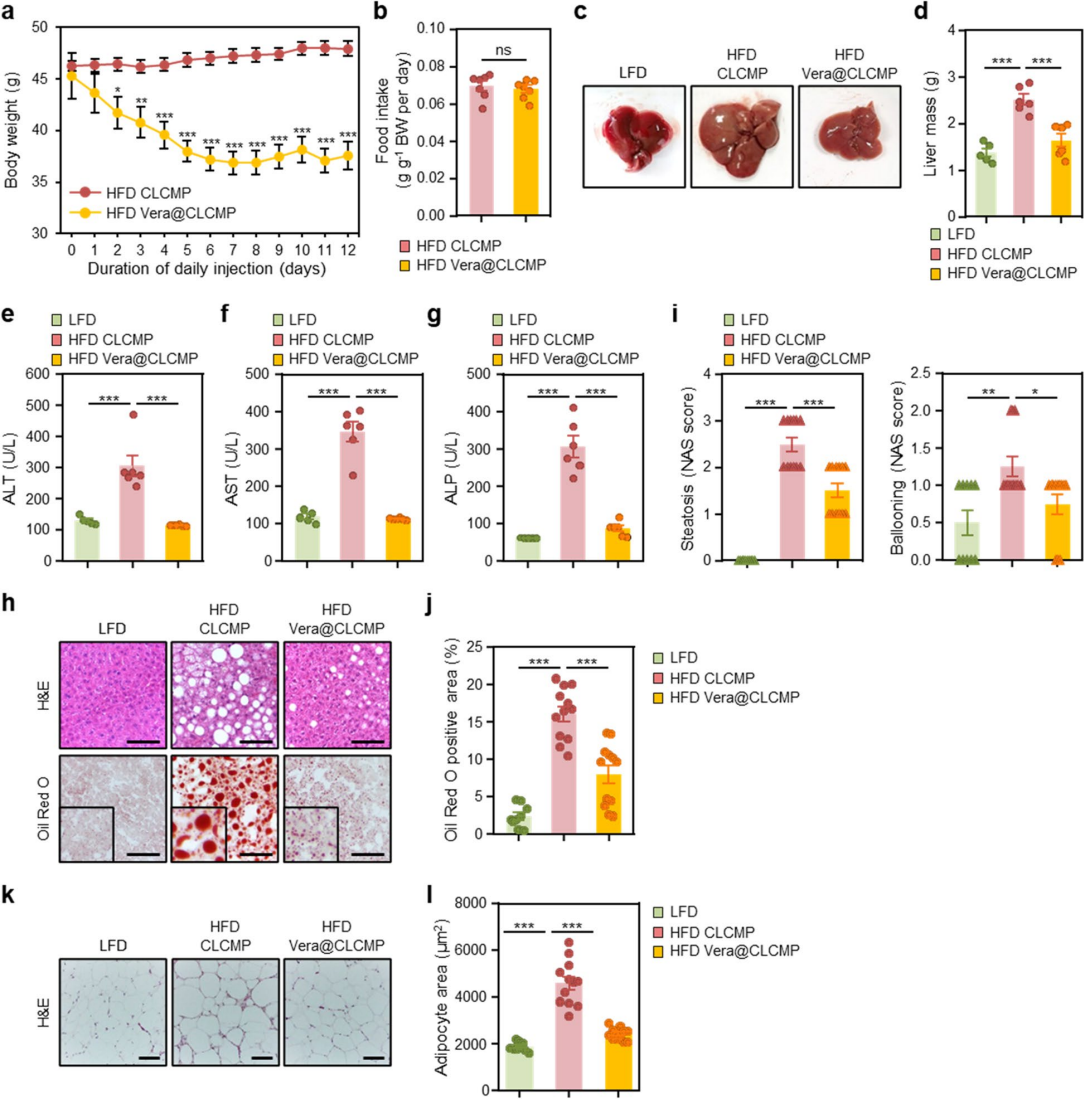
Vera@CLCMP patches reverse diet-induced obesity and hepatic steatosis. **A-L** C57BL/6 male mice kept on HFD for 9 weeks had Vera@CLCMP or CLCMP patches applied to the dorsal skin. LFD-fed mice of the same age were used as a negative control. **A** Body weight of mice fed a HFD and treated with Vera@CLCMP or CLCMP patches. **B** Food intake during treatment period. **C** Gross liver morphology and **D** total liver mass of mice in each group indicated. **E-G** Serum ALT, AST, and ALP levels. **H** H&E staining (upper) and Oil-Red O staining (lower) of liver sections from mice in each group indicated. Scale bar, 100 μm. **I** Histological MAFLD activity score (NAS). **J** Oil-Red O-stained area was quantified. **K** H&E staining of epididymal white adipose tissue from mice in each group indicated. Scale bar, 100 μm. **L** Average adipocyte area of epididymal white adipose tissue was quantified. Data is shown as mean ± SEM. *p < 0.05; **p < 0.01; ***p < 0.001 (One-way ANOVA, followed by Tukey’s test)

To investigate the potential anti-obesity effects of Vera@pullulan patches, we fed HFD to C57BL/6 mice and applied 50 mg/kg Vera@pullulan or pullulan patches to their dorsal skin 3 times per week for 2 weeks, while monitoring their body weight. Vera@pullulan patch-treated obese mice showed a decrease in body weight with no effect on food intake (Additional file 1 Fig. S6a, b). We then applied 50 mg/kg Vera@CLCMP or CLCMP patches to the dorsal skin of HFD-fed C57BL/6 mice one time per week for 2 weeks. Treatment with Vera@CLCMP patches resulted in a 7.7-g weight loss in HFD-fed obese mice after only 1 week of administration, with no further effect thereafter (Fig. 7a), whereas food intake was similar between the two groups of obese mice treated with vehicle and Vera@CLCMP patches (Fig. 7b). These results suggest that Vera@CLCMP patches with a low dosing frequency are sufficient to reduce body weight in obese mice.

Macroscopically, while the liver of vehicle-treated obese mice was pale, the liver of Vera@CLCMP patch-treated obese mice was a reddish-brown color (Fig. 7c). Vera@CLCMP patch-treated obese mice consistently exhibited a remarkable reduction in liver weight compared to vehicle-treated obese mice (Fig. 7d). The serum levels of ALT, AST, and ALP were also significantly lower after treatment with Vera@CLCMP patches (Fig. 7e-g). We then histologically stained the livers of Vera@CLCMP patch-treated obese mice with hematoxylin and eosin and Oil Red O. Vehicle-treated HFD-fed mice presented signs of hepatocellular ballooning, lobular inflammation, and steatosis. However, treatment with Vera@CLCMP patches resulted in a significant reduction in hepatocellular steatosis, ballooning, and lobular inflammation compared to vehicle-treated HFD-fed mice (Fig. 7h, i). Oil Red O staining analysis revealed a dramatic reduction in hepatic lipid levels in Vera@CLCMP patch-treated HFD-fed mice compared to vehicle-treated HFD-fed mice, which was consistent with the results of hematoxylin and eosin staining (Fig. 7h, j). The adipocyte size of the epididymal white adipose tissue from Vera@CLCMP patch-treated mice was significantly smaller than that of vehicle-treated mice after HFD feeding (Fig. 7k, l). These results suggest that treatment with Vera@CLCMP patches protects against diet-induced obesity and hepatic steatosis.

### Vera@CLCMP patches attenuate accumulation of ubiquitinated proteins and NLRP3 inflammasome in diet-induced obese mice

**Fig. 8:**
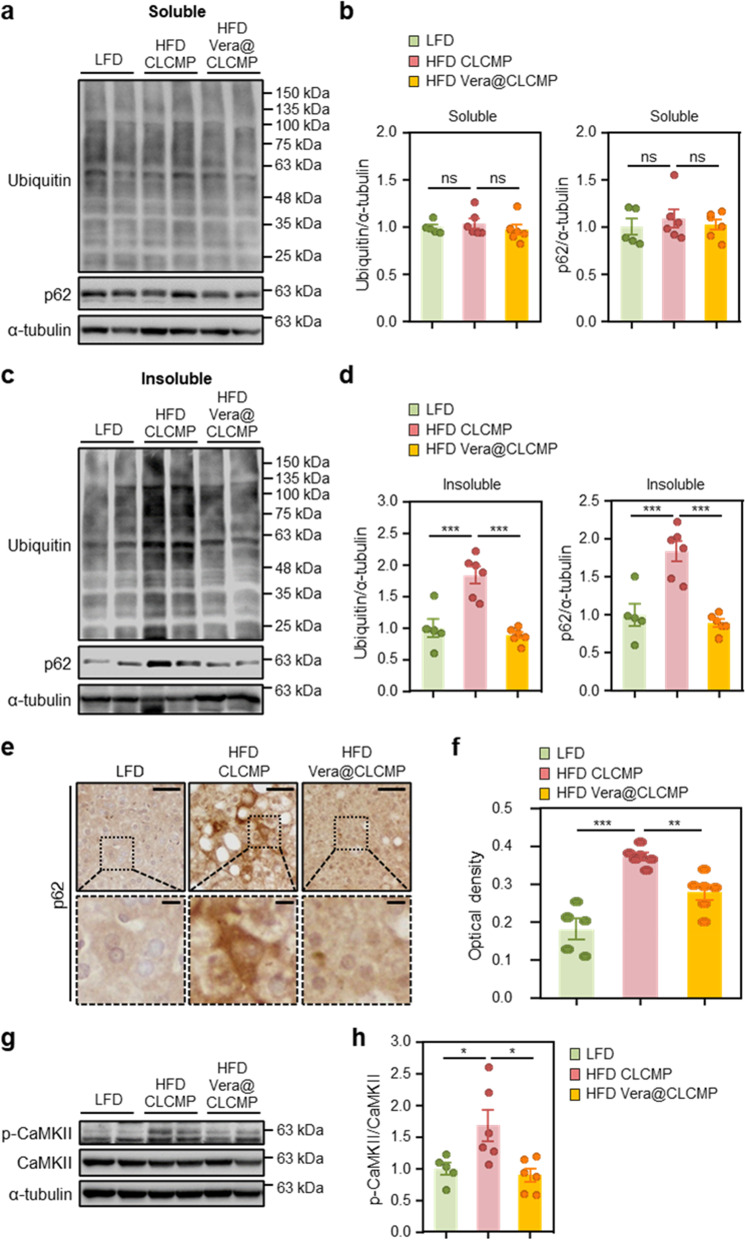
Vera@CLCMP patches improve the accumulation of ubiquitinated proteins by inhibiting the activation of CaMKII. **A-H** C57BL/6 male mice kept on HFD for 9 weeks had Vera@CLCMP or CLCMP patches applied to the dorsal skin. LFD-fed mice of the same age were used as a negative control. **A, C** Triton X-100-soluble and-insoluble fractions of liver tissue lysates were immunoblotted with anti-ubiquitin and anti-p62 antibodies. α-tubulin served as a loading control. **B, D** Band intensities were quantified and normalized to control levels. **E** Immunohistochemical staining for p62 in liver tissues from mice in each group. Boxed areas are magnified in the bottom panels. Scale bars, 50 μm; 10 μm (insets). **F** Optical density of p62 immunoreactivity. **G** Liver tissues were collected from mice in each group and analyzed by immunoblotting with anti-phospho-CaMKII antibody. α-tubulin served as a loading control. **H** Band intensities were quantified and normalized to the CaMKII intensities. Data is shown as mean ± SEM. *p < 0.05; **p < 0.01; ***p < 0.001; ns, not significant (One-way ANOVA, followed by Tukey’s test)

We explored the molecular mechanisms underlying the therapeutic effects of Vera@CLCMP patches in HFD-induced hepatic steatosis. Immunoblotting and immunohistochemistry were used to investigate the effect of Vera@CLCMP patches on autophagy clearance in the liver of HFD-fed mice. There was no significant difference in the levels of ubiquitinated proteins and p62 in detergent-soluble fractions from the livers of Vera@CLCMP patch-treated HFD-fed mice and vehicle-treated HFD-fed mice (Fig. 8a, b). Ubiquitinated proteins and p62 levels in detergent-insoluble fractions were elevated in HFD-fed mouse liver tissues. This increase was suppressed in HFD-fed mice treated with Vera@CLCMP patches (Fig. 8c, d), indicating a reduction in HFD-induced protein inclusion formation.

Immunohistochemical analysis revealed that p62 staining in the cytoplasm of hepatocytes was significantly reduced in Vera@CLCMP patch-treated HFD-fed mice compared to vehicle-treated HFD-fed mice (Fig. 8e, f). To explore whether treatment with the Vera@CLCMP patch regulates CaMKII activity, which is highly sensitive to cytosolic Ca^2+^ levels in hepatocytes during obesity [38], we examined the phosphorylation levels of CaMKII in the livers of HFD-fed mice. The results of immunoblot analysis indicated that CaMKII phosphorylation was significantly downregulated in Vera@CLCMP patch-treated HFD-fed mice compared to vehicle-treated HFD-fed mice (Fig. 8g, h), suggesting that the CaMKII signaling pathway is involved in the therapeutic benefits of the Vera@CLCMP patch in hepatic steatosis.

**Fig. 9:**
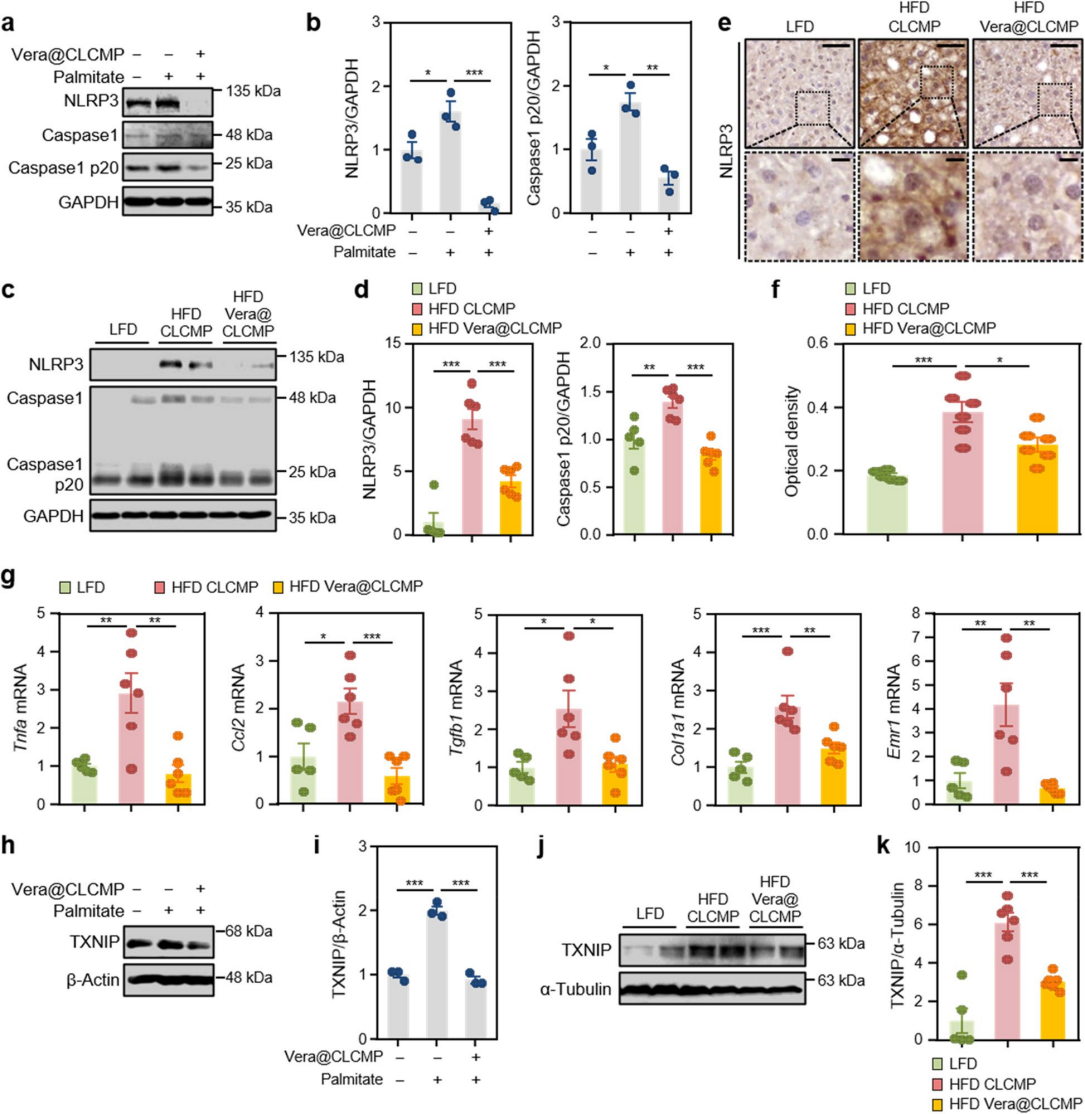
Vera@CLCMP patches ameliorate NLRP3-inflammasome activation by inhibiting the expression of TXNIP. **A, B, H, I** HepG2 cells were treated with 100 μM palmitate or BSA (vehicle) for 24 h in the presence or absence of a release medium incubated with 38.35 μg/mL Vera@CLCMP at 37 °C for 6 days. Cell lysates were immunoblotted with anti-NLRP3 and anti-caspase-1 (**A**) or anti-TXNIP (**H**) antibodies. GAPDH or α-tubulin served as a loading control. **B, I** Band intensities were quantified and normalized to control band intensities. **C-G, J, K** C57BL/6 male mice kept on HFD for 9 weeks had Vera@CLCMP or CLCMP patches applied on their dorsal skin. LFD-fed mice of the same age were used as a negative control. **C** Liver tissue lysates were immunoblotted with anti-NLRP3 and anti-caspase-1 antibodies. GAPDH served as a loading control. **D** Band intensities were quantified and normalized to control band intensities. **E** Immunohistochemical staining for NLRP3 in liver tissues from mice in each group indicated. Boxed areas are magnified in the bottom panels. Scale bars, 50 μm; 10 μm (insets). **F** Optical density of NLRP3 immunoreactivity. **G** qRT-PCR analysis of Tnfa, Ccl2, Tgfb1, Col1a1, and Emr1 mRNA levels in liver tissues from mice in each group indicated. **J** Liver tissue lysates were immunoblotted with anti-TXNIP antibody. α-tubulin served as a loading control. **K** Band intensities were quantified and normalized to control band intensities. Data is shown as mean ± SEM. *p < 0.05; **p < 0.01; ***p < 0.001 (One-way ANOVA, followed by Tukey’s test)

To evaluate whether the administration of Vera@CLCMP patches influences hepatic NLRP3 inflammasome activation, we measured the expression levels of NLRP3 inflammasome markers in palmitate-induced HepG2 cells and the livers of HFD-fed mice. NLRP3 and cleaved caspase-1 protein levels were increased by palmitate treatment, which was reversed by the release medium of Vera@CLCMP patches (Fig. 9a, b). Transdermal administration of Vera@CLCMP patches decreased NLRP3 and cleaved caspase-1 protein levels in the HFD-fed mouse liver (Fig. 9c, d). Immunohistochemical analysis also revealed that the transdermal administration of Vera@CLCMP patches in the cytoplasm of hepatocytes significantly reduced NLRP3 expression (Fig. 9e, f). Consistent with the protein expression data, quantitative real time-PCR (qRT–PCR) showed significantly lower mRNA expression of *Nlrp3*, *Casp1*, and *Il-1β* in the livers of Vera@CLCMP patch-treated HFD-fed mice than in vehicle-treated HFD-fed mice (Additional file 1 Fig. S7). We further measured the expression of inflammatory genes in the liver of LFD-fed, HFD-fed, and Vera@CLCMP patch-treated HFD-fed mice using qRT-PCR. Mice treated with Vera@CLCMP patches had a lower expression of several inflammatory genes, including *Tnfa*, *Ccl2* (also known as *Mcp1*), *Tgfb1*, *Col1a1*, and *Emr1* (also known as F4/80), compared to vehicle-treated control mice (Fig. 9g).

TXNIP appears to be involved in NLRP3 inflammasome activation and MAFLD pathogenesis [21, 26, 39]. Therefore, we evaluated whether administration of Vera@CLCMP patches affected TXNIP expression in palmitate-treated HepG2 cells and HFD-fed mouse livers. TXNIP protein expression levels were found to be reduced after pretreatment with the release medium of Vera@CLCMP patches (Fig. 9h, i). Similarly, HFD-fed mice treated with Vera@CLCMP patches had substantially decreased TXNIP protein expression levels compared to vehicle-treated control mice (Fig. 9j, k). Overall, the findings suggest that transdermal administration of Vera@CLCMP patches can suppress hepatic TXNIP/NLRP3 pathways during HFD-induced MAFLD.

## Discussion

Ca^2+^ channel blockers have been widely used to treat hypertension, chest pain, and arrhythmias. We recently discovered that intracellular Ca^2+^ homeostasis in hepatocytes is disrupted in HFD-induced mice, resulting in insufficient autophagosome clearance, which was ameliorated by Ca^2+^ channel blocker treatment [24, 25]. However, because of its high toxicity and low bioavailability, clinical applications of verapamil in obesity-related diseases have not been successfully developed. Here, we developed a CLCMP-based hydrogel patch for the efficient and sustained transdermal delivery of verapamil, and cell viability assay and Kaplan–Meier survival analysis revealed that Vera@CLCMP patches were much less toxic to hepatocytes and mice. Increasing evidence suggests that obesity and sustained insulin resistance are accompanied by a chronic low-grade inflammatory state, also known as metabolic inflammation, in the metabolic tissues [40]. The NLRP3 inflammasome has been linked to the etiology of several inflammatory and metabolic diseases, including MAFLD and type 2 diabetes [41–43]. In line with this concept, obesity-induced insulin resistance is alleviated in mice genetically deficient in NLRP3 [44, 45]. In the present study, we showed that sustained transdermal delivery of verapamil via CLCMP-based patches could reduce insulin resistance and MAFLD by inhibiting the TXNIP/NLRP3 inflammasome pathway and improving autophagic degradation of protein aggregates.

Pullulan is a natural extracellular polysaccharide produced by the yeast-like fungus *Aureobasidium pullulans* [46]. Pullulan is becoming more popular in the biomedical field due to its non-irritant, non-toxic, non-immunogenic, biodegradable, and skin-friendly properties, with excellent patch-forming features and adhesive properties [47–49]. Nevertheless, some attributes of pure pullulan patches, such as their highly hydrophilic nature and consequent weakness and poor flexibility, limit their widespread use in clinical settings [50]. As pullulan is easily chemically modified due to its abundance of hydroxyl groups [46], more research is needed to develop new advanced functions and broaden its applicability. Therefore, we developed a strategy using a structure in which the methoxy group of pullulan was derivatized by carboxylation. Our results clearly demonstrated that the CLCMP-based hydrogel patch has a more flexible and ductile structure than the pullulan patch, with improved barrier performance and enhanced loading capacity. Moreover, we demonstrated that the crosslinking of CMP with citric acid and glycerol can enhance sustained drug delivery and increase the bioavailability of verapamil.

Hydrogels are hydrophilic 3D polymeric networks capable of absorbing a large amount of water. In general, the fabrication of hydrogel DDS needs to maintain the drug bioactivity, and through packaging, transport and storage, both the drug and the hydrogel must be chemically and physically stable. In this reason, chemical binding interactions between verapamil and hydrogel are not introduced as explained in Fig. 2d. When verapamil-loaded hydrogel was exposed on air, hydrogel absorbed water from air (normal humidity = 50% at 25 °C, 1 atm, calculated amount of water = 9.98 g/Kg of air).

After absorbing water from the air, verapamil can release and penetrate the skin through transepidermal and transappendageal pathways. The transepidermal pathway delivers verapamil through the stratum corneum. Intracellular penetration occurs either through corneocytes, which allow the transport of hydrophilic substances, or through intercellular space which allows the diffusion of lipophilic substances across lipid membranes. The transappendageal route involves the passage of verapamil through the hair follicles and sweat glands. Thus, topical application of verapamil in the form of patches can release verapamil into the skin and into the systemic circulation through percutaneous absorption, percutaneous penetration during which the verapamil transports from the surface of the stratum corneum through the skin to the systemic circulation, and through diffusion or skin penetration of verapamil through the pores [51].

We and others previously reported that Ca^2+^ channel blockers can reduce mouse body weight [24, 52]. Fat can be reduced by nifedipine, a Ca^2+^ channel blocker, in obese mice [25, 53]. There is an increase in fatty acid synthase (FAS) gene expression and triglyceride accumulation in the adipose tissue of obese rodents [54, 55]. Interestingly, treatment with Ca^2+^ channel blockers has been indicated to inhibit FAS expression [53, 56]. Moreover, nifedipine can prevent a decrease in the core temperature of obese mice [53], which is associated with the increase in body fat use to compensate for energy expenditure. Yoshida et al. reported that treatment of obese mice with a Ca^2+^ channel blocker, benidipine, resulted in the activation of brown adipose tissue to induce weight loss [52]. It is therefore possible that weight loss in Vera@CLCMP patch-treated obese mice may be partially explained by the regulatory effect on fat storage, FAS activity, and thermogenesis via an intracellular Ca^2+^-dependent mechanism.

Drug-induced hepatotoxicity is common and can be caused by immune-mediated mechanisms and various medications, often due to the direct toxicity of the parent drug or its metabolites [57]. Overdoses of immediate-release Ca^2+^ channel blockers can result in hypotension, bradycardia, and cardiac arrest [58]. Although liver injury caused by Ca^2+^ channel blockers is not a common side effect and is reversible, severe symptoms have been reported infrequently. Our results demonstrate that CLCMP patches provide long-term delivery of verapamil, which results in a decrease in dosing frequency, and consequently the minimization of the associated side effects. Similarly, estradiol transdermal patches reduce side effects, including liver damage, compared to its oral formulations [59].

The NLRP3 inflammasome in the liver is activated in the pathogenesis of various metabolic liver diseases [60, 61], triggering caspase-1 activation, which produces the main proinflammatory cytokines. All of these processes are associated with MAFLD development [62]. Consistent with this finding, we found that NLRP3 and cleaved caspase-1 expression levels were increased in both palmitate-treated HepG2 cells and HFD-fed mouse livers. The hepatic expression levels of inflammasome components are associated with the degree of liver injury and fibrosis, which are caused by pyroptotic cell death [63]. Lipotoxic hepatocytes can activate Kupffer cells, the resident hepatic macrophages that cause inflammation [60]. Moreover, cholesterol crystals formed by apoptotic hepatocytes are known to play an important role in the activation of NLRP3 inflammasomes in Kupffer cells, resulting in the production of proinflammatory cytokines [64]. Therefore, it is possible that non-parenchymal cells were also involved in NLRP3 activation in the liver of HFD-fed mice. Given that Ca^2+^ channel blockers can inhibit proinflammatory cytokine release by Kupffer cells, which are activated by high levels of intracellular Ca^2+^ [65, 66], the observed effects of Vera@CLCMP patch administration on HFD-induced hepatic steatosis may be mediated by proinflammatory cytokines released from non-parenchymal cells.

## Conclusion

In summary, this study presents the development of an effective CLCMP-based patch for the transdermal delivery of verapamil. CLCMP-based patches showed a higher loading capacity and increased release control than pure pullulan patches. Upon application to the skin, verapamil is released sustainably from the CLCMP-based patch and subsequently penetrates the stratum corneum, reaching the dermis. By inhibiting the TXNIP/NLRP3 pathways and improving autophagic clearance, transdermal application of Vera@CLCMP patches alleviated obesity-induced insulin resistance and hepatic steatosis. With its simple fabrication process and wide applicability, Vera@CLCMP patches not only improve bioavailability but also introduce an enhanced route of administration for verapamil, improving patient compliance. Our findings offer a valuable clinical tool for the simple, efficient, and safe administration of verapamil to treat metabolic syndrome.

## Supplementary Information

**Additional file 1:**

## Data Availability

The datasets used and/or analyzed during the current study are available from the corresponding author on reasonable request.
