# Supplementary material for: Verapamil-loaded supramolecular hydrogel patch attenuates metabolic dysfunction-associated fatty liver disease via restoration of autophagic clearance of aggregated proteins and inhibition of NLRP3
Source: Biomater Res. 2023 Jan 20;27:4. doi: 10.1186/s40824-023-00342-5 (PMC9854054; doi:10.1186/s40824-023-00342-5)
Supplement: Supplementary file 1 — Additional file 1: [file 40824_2023_342_MOESM1_ESM.docx]

RESEARCH ARTICLE

**Supporting Information**

Verapamil-loaded supramolecular hydrogel patch attenuates metabolic dysfunction-associated fatty liver disease via restoration of autophagic clearance of aggregated proteins and inhibition of NLRP3

Do Kyung Kim^a^, Daewon Han^b^, Jeongyun Bae^b^, Haeil Kim^b^, Solji Lee^b^, Jong-Seok Kim^c^, Young-Gil Jeong^a^, Jongdae Shin^b, c^, and Hwan-Woo Park^b, c*^


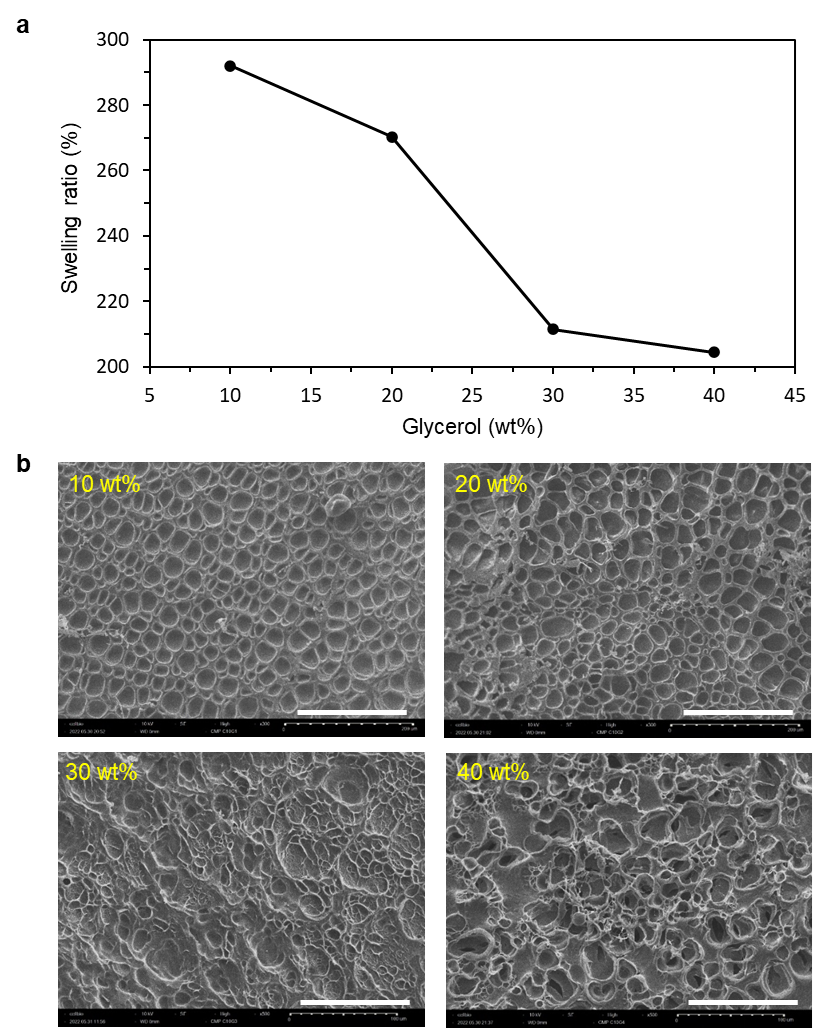


**Fig. S1. a** Swelling ratio depending on adding amount of glycerol. **b** SEM micrographs of the freeze-dried samples of 10-40 wt% glycerol and 10 wt% citric acid. Scale bar, 200 µm.


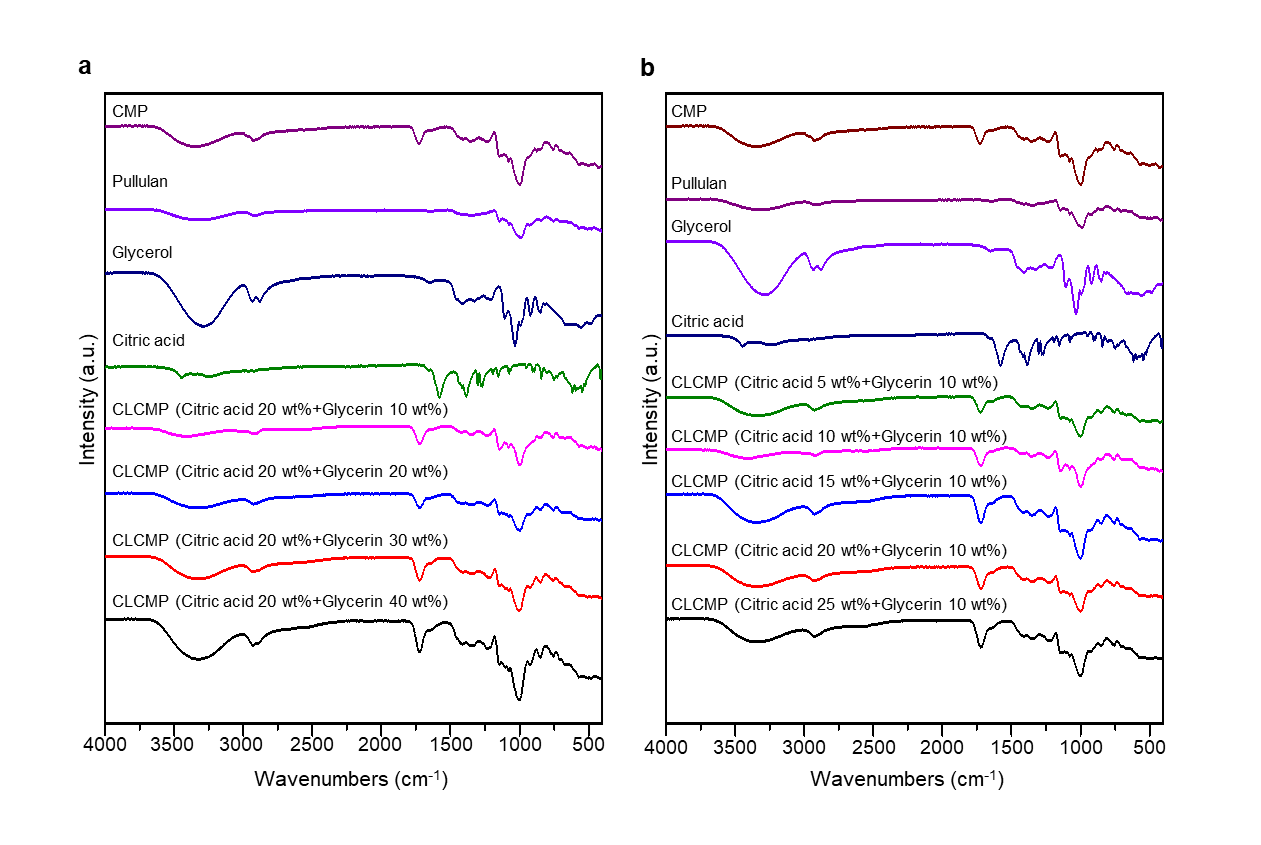


**Fig. S2.** **a** FTIR spectra of CMP, pullulan, glycerol, citric acid, CLCMP prepared with 10-40 wt% glycerol and 10 wt% citric acid and **b** 5-25 wt% citric acid and 10 wt% glycerol.


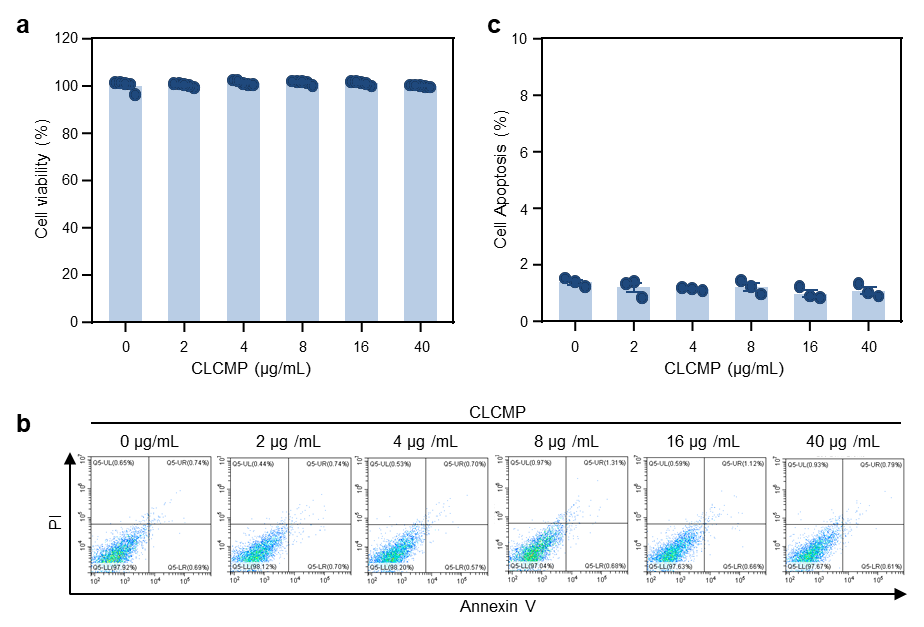


**Fig. S3.** *In vitro* cytotoxicity of CLCMP patches. **a-c** HepG2 cells were treated with the indicated concentration of CLCMP for 24 h. **a** Cell viability was measured by WST-8 assay. **b** HepG2 cells were stained with annexin V-FITC and PI and then analyzed for apoptosis by flow cytometry. The percentage of apoptotic HepG2 cells are shown in **c**. Data is shown as mean ± SEM. ns, not significant (One-way ANOVA, followed by Tukey’s test).


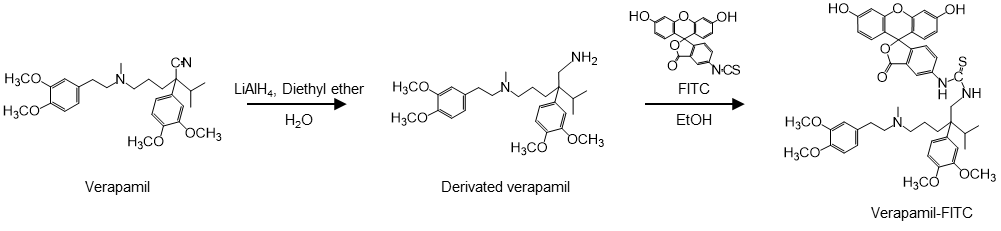


**Fig. S4.** Chemical structures schemes of the formation of verapamil-FITC conjugate.

**
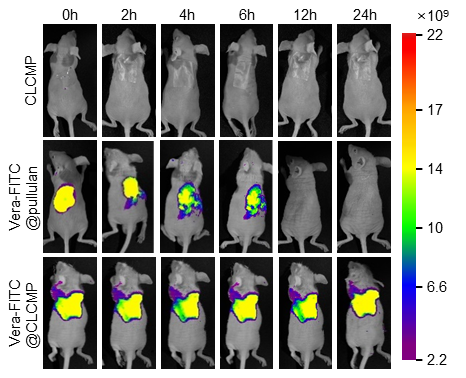
**

**Fig. S5.** *In vivo* imaging of Vera-FITC@CLCMP patches in mice. Vera-FITC@pullulan and Vera-FITC@CLCMP patches were applied to the dorsal skin of hairless mice. Fluorescence imaging of mice at 2, 4, 6, 12, and 24 h after treatment with CLCMP, Vera-FITC@pullulan, or Vera-FITC@CLCMP patches.


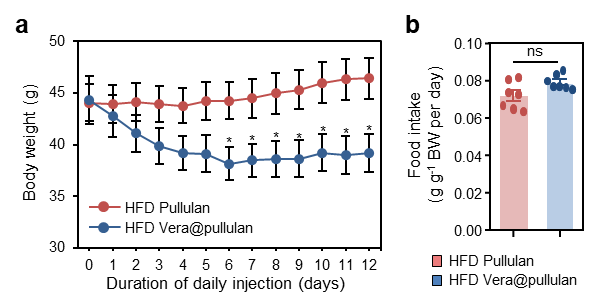


**Fig. S6.** Effect of Vera@pullulan patches on mice body weight and food intake. C57BL/6 male mice kept on HFD for 9 weeks were applied with Vera@pullulan or pullulan patches on the dorsal skin. **a** Body weight of mice fed a HFD and treated with Vera@pullulan or pullulan patches. **b** Food intake during treatment period. Data is shown as mean ± SEM. *p < 0.05; ns, not significant (Student’s *t*-test).


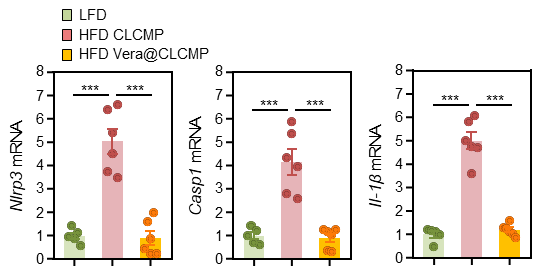


**Fig. S7.** C57BL/6 male mice kept on HFD for 9 weeks were applied with Vera@CLCMP or CLCMP patches on the dorsal skin. LFD-fed mice of the same age were used as a negative control. Liver tissues were collected from mice in each group indicated. Relative mRNA expression levels of *Nlrp3*, *Casp1*, and *Il-1β* were determined by qRT-PCR. Data are shown as mean ± SEM. ***p < 0.001 (One-way ANOVA, followed by Tukey’s test).

**Table S1. List of primer pairs used for qRT-PCR analysis.**

| **Gene** | **Forward Primer (5’-3’)** | **Reverse Primer (5’-3’)** |
| --- | --- | --- |
| GAPDH | AAGGTCATCCCAGAGCTGAA | CTGCTTCACCACCTTCTTGA |
| Nlrp3 | AGCCTTCCAGGATCCTCTTC | CTTGGGCAGCAGTTTCTTTC |
| Casp1 | AGATGGCACATTTCCAGGAC | GATCCTCCAGCAGCAACTTC |
| Il-1β | TCTTTGAAGTTGACGGACCCCC | TGAGTGATACTGCCTGCCTG |
| Tnfa | TCCCAGGTTCTCTTCAAGGGA | GGTGAGGAGCACGTAGTCGG |
| Ccl2 | CATCCACGTGTTGGCTCA | GATCATCTTGCTGGTGAATGAGT |
| Tgfb1 | CTCCCGTGGCTTCTAGTGC | GCCTTAGTTTGGACAGGATCTG |
| Col1a1 | GCTCCTCTTAGGGGCCACT | CCACGTCTCACCATTGGGG |
| Emr1 | CCCCAGTGTCCTTACAGAGTG | GTGCCCAGAGTGGATGTCT |
